# Trilayered Gires–Tournois Resonator with Ultrasensitive Slow-Light Condition for Colorimetric Detection of Bioparticles

**DOI:** 10.3390/nano13020319

**Published:** 2023-01-12

**Authors:** Jiwon Kang, Young Jin Yoo, Joo Hwan Ko, Abdullah Al Mahmud, Young Min Song

**Affiliations:** 1 School of Electrical Engineering and Computer Science, Gwangju Institute of Science and Technology, 123 Cheomdangwagi-ro, Gwangju 61005, Republic of Korea; 2 Anti-Viral Research Center, Gwangju Institute of Science and Technology, 123 Cheomdangwagi-ro, Gwangju 61005, Republic of Korea; 3 Artificial Intelligence (AI) Graduate School, Gwangju Institute of Science and Technology, 123 Cheomdangwagi-ro, Gwangju 61005, Republic of Korea

**Keywords:** Gires–Tournois resonator, slow-light effect, colorimetric sensing, bioparticle detection, porous structure

## Abstract

Over the past few decades, advances in various nanophotonic structures to enhance light–matter interactions have opened numerous opportunities for biosensing applications. Beyond the successful development of label-free nanophotonic biosensors that utilize plasmon resonances in metals and Mie resonances in dielectrics, simpler structures are required to achieve improved sensor performance and multifunctionality, while enabling cost-effective fabrication. Here, we present a simple and effectual approach to colorimetric biosensing utilizing a trilayered Gires–Tournois (GT) resonator, which provides a sensitive slow-light effect in response to low refractive index (RI) substances and thus enables to distinguish low RI bioparticles from the background with spatially distinct color differences. For low RI sensitivity, by impedance matching based on the transmission line model, trilayer configuration enables the derivation of optimal designs to achieve the unity absorption condition in a low RI medium, which is difficult to obtain with the conventional GT configuration. Compared to conventional bilayered GT resonators, the trilayered GT resonator shows significant sensing performance with linear sensitivity in various situations with low RI substances. For extended applications, several proposed designs of trilayered GT resonators are presented in various material combinations by impedance matching using equivalent transmission line models. Further, comparing the color change of different substrates with low RI NPs using finite-difference time-domain (FDTD) simulations, the proposed GT structure shows surpassing colorimetric detection.

## 1. Introduction

For decades, numerous nanophotonic biosensors have emerged to address the limitations of current bioanalytical methods in terms of sensitivity, throughput, ease of use, and miniaturization [1,2,3]. Various nanophotonic structures that control subwavelength volume light and enhance light–matter interactions open interesting prospects for biosensing. Especially, label-free nanophotonic biosensors, such as evanescent field-based sensing along with the plasmon resonance of metals and Mie resonance of dielectrics, have been continuously developed for enhanced interactions between the probing light and the analyte [4,5,6,7,8,9,10,11,12]. Because these nanophotonic structures rely on the iterative patterning of engineered nanostructures, electron beam or focused ion beam lithography is commonly used due to their flexibility to pattern various nanostructures [11,12,13,14,15,16,17]. Meanwhile, recent advances in nanostructures, on-chip, optoelectronic integration, and data science toolkits are required to achieve improved sensor performance and multifunctionality, and thus simpler structures are pursued for flat integration enabling smaller footprints and portability [18,19].

Highly absorbent photonic structures have recently received much attention due to their potential applications in solar cells [20,21,22], electro-optic detectors [23,24], and heat emitters [25,26,27,28]. In particular, Gires–Tournois (GT) resonators composed of thin films on a reflective metal mirror have been successfully demonstrated to promote ultrahigh absorption and strong light–matter interactions with multibeam interference and a slow-light effect [29,30,31,32,33,34,35]. This geometry exhibits numerous advantages, such as simple fabrication, facile spectral tuning, omnidirectional strong absorption, and the possibility of active modulation of absorption. Based on these promising properties, considerable studies have been conducted to explain optical properties, such as the conditions for the ultrahigh absorption of these systems using impedance or admittance matching analysis or assuming a constant tangential electric or magnetic field [33,34,36]. Recently, GT resonators using nonlinear effective phase change have been widely studied as planar metasurfaces, including dynamic phase change absorbers, achromatic surface clocking and lenses, and colored perovskite solar cells [32,33,34,35,37]. Nevertheless, these powerful resonators, capable of various media combinations, have still not been optimized in unique conditions for strong absorption with sensitive slow-light for biosensing applications, such as low refractive index (RI) virus detection.

Here, we present a clever strategy for the colorimetric detection of bioparticles using a modified GT resonator for resonant responses sensitive to low RI substance on the surface. For a strong light–matter interaction even at a low RI, we employed trilayer configurations of GT resonators with a combination of lossless/lossy media for scalable designs with lower RIs due to porosities (*P_r_*). For extended applicability to various target analytes of trilayer configurations, we establish a design rule by impedance matching using equivalent models of trilayered GT resonators based on the transmission line theory [38,39,40]. As a practical application, the optimal design for low RI bioparticles is evaluated by comparing group delay (GD) values among several candidate designs. With an optimal design, the trilayered GT resonator has the advantage of comparing the optical properties in several situations of low-index substances with bilayered GT resonators composed of lossless medium and lossy medium. The trilayered GT resonator designed for low RI bioparticle detection is characterized through spatial and spectral reflectance distribution and RGB color representation from the calculation results according to the gap, size, and RI of nanoparticles. For experimental confirmation, we fabricate a trilayered GT resonator by glancing angle deposition (GLAD). After surface treatment for immunoassay, the trilayered GT resonator is demonstrated for colorimetric detection using bioparticles functionalized with antigen on the surface.

## 2. Materials and Methods

### 2.1. Optical Calculation

To achieve optical characteristics, including the amplitude, phase, and GD of the reflected light from the GT resonator, the whole optical calculation was conducted by FDTD method in the commercial software (FullWAVE, RSoft Design Group, Synopsys, Mountain View, CA, USA). The physical formation of NPs was reconstructed in the computer-aided design (CAD), and the unpolarized visible light (wavelength in the range of 400 nm to 800 nm) was launched at a normal angle. Additionally, entire structures were aligned in the Euclidean coordinates, and material dispersions and extinction coefficients were considered for realistic simulation. The configuration for simulation was set with a continuous plane wave and a grid size of 5 nm. The customized MATLAB code was developed to translate the reflectance regarding visible wavelength into the emerged color from a GT resonator and derive the effective complex RI of *P_r_* thin film using volume averaging theory from bulk thin film.

### 2.2. Fabrication of GT Resonator

Before the 100 nm thick Au reflector deposition, single-side polished silicon (100) wafer was sonicated and washed with acetone, isopropanol, and deionized (DI) water in that order and then dried using a nitrogen gun. By using electron beam evaporation (KVE-E2000, Korea Vacuum Tech, Gimpo, Republic of Korea) at a rate of ~ 1 Å s^−1^ under high vacuum conditions (approximately 10^−6^ Torr), a 60 nm thick *P_r_*–Ge thin film was deposited on an Au reflector with identical conditions. The *P_r_*–Ge layer was deposited by the GLAD processes after being embedded on a customized slanted sample holder at the deposition angle of 70° to control the RI of the lossy medium. For a uniform surface, the substrate was flipped upside down at half of the target thickness; then, a 180 nm thick SiO_2_ layer was deposited by plasma-enhanced chemical vapor deposition (PECVD, System 100, Oxford, Bristol, UK) using RF plasma (170 sccm of SiN_4_ gas, 710 sccm of N_2_O gas, 20 W, 0.1 Torr, 90 s, 150 °C).

### 2.3. Fixing NPs on the GT Resonator

In order to fix NPs (100 nm diameter SiO_2_ particles, Sigma-Aldrich, St. Louis, MO, USA) on GT resonator, both were biofunctionalized with antigen (Anti-Spike-RBD-hIgG1, InvivoGen, San Diego, CA, USA) and antibody (Spike-RBD-His, InvivoGen, USA). After synthesis, NPs were diluted in premade phosphate-buffered saline (PBS, Biosesang Co., Ltd., Seongnam, Republic of Korea) to obtain the desired concentration of 10 ng mL^−1^. Using a gastight microsyringe (Legato 210, KD scientific Inc., Holliston, MA, USA), a 300 nL volume solution was dropped onto the GT resonator. The NPs were rotated in the evaporating droplet due to the Marangoni flow followed by rinsing with PBS solution for 30 s and DI water for 10 s to block nonspecific binding and rinse the impurities.

### 2.4. Optical Microscope (OM) Images

The completed GT resonator with NPs was mounted onto the stage of optical microscopy system (BX53M, Olympus, Tokyo, Japan). Optical bright-field micrographs were captured using a 100× objectives lens (MPlanFLN, Olympus, Japan) and a computer-connected CMOS camera (STC-MCCM200U3V, Omron Sentech, Ebina, Japan) under a white LED lamp as a continuous light source.

### 2.5. SEM Images

The ultrahigh-resolution field-emission scanning electron microscope (UHR FE-SEM, Verios 5 UC, ThermoFisher, Waltham, MA, USA) was utilized to validate the existence of NPs so that OM raw image was matched to the SEM image. Because the surfaces of GT resonator and NPs are made of SiO_2_, 3 nm thick platinum was plated on the samples using high-vacuum sputter coater (EM ACE600, Leica, Wetzlar, Germany). All SEM micrographs were obtained at an acceleration voltage of 10 kV with a beam current of 25 pA.

### 2.6. TEM Images

To acquire the cross-sectional image of *P_r_*–Ge, the GT resonator was thinned using a focused ion beam system (FIB, Ethos NX5000, Hitachi, Tokyo, Japan). The TEM (Tecnai G 530 S-Twin, FEI Co., Hillsboro, OR, USA) was used to investigate the prepared specimen.

### 2.7. Customized Circle Annotator

For annotation, a homemade semiautomatic annotator was used to extract the physical position of the NPs in SEM images. The circle Hough Transform from OpenCV-Python was adopted to build the annotator. By handling the five variations for circle identification, it was possible to easily edit the annotation and erase the wrong annotation.

## 3. Results

### 3.1. Comparison of Different GT Resonators with Bi-/Trilayer Configurations

**Figure 1:**
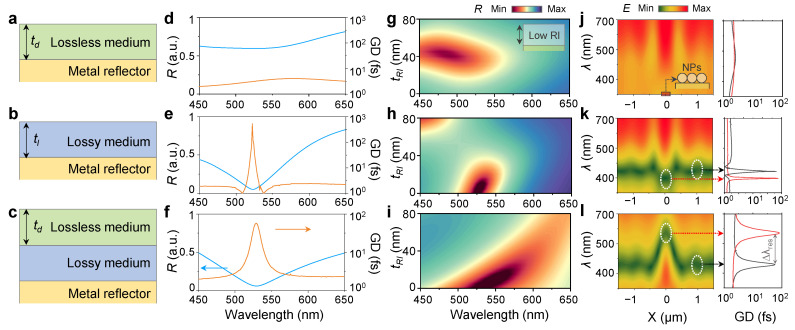
(**a**–**c**) Schematic of Gires–Torunois (GT) resonators with bilayers of each (**a**) lossless medium and (**b**) lossy medium and (**c**) trilayer configurations of lossless and lossy media. (**d**–**f**) Reflectivity (*R*, blue) and group delay (GD, orange) of GT resonators with bilayers of each (**d**) lossless medium and (**e**) lossy medium and (**f**) trilayer configurations of lossless and lossy media. (**g**–**i**) Reflectivity contour plot for thickness (*t_RI_*) variation of low refractive index (RI) layer on GT resonators with bilayers of each (**g**) lossless medium and (**h**) lossy medium and (**i**) trilayer configurations of lossless and lossy media. (**j**–**l**) Spatial and spectral distributions of electric field (*E*) and GD with GT resonators with bilayers of each (**j**) lossless medium and (**k**) lossy medium and (**l**) trilayer configurations of lossless and lossy media.

Figure 1 demonstrates a comparison of optical characteristics for different configurations of GT resonators with bilayer and trilayer configurations. In the bilayer configurations, each lossless medium and lossy medium can be designed on a metal reflector (Figure 1a,b). In the bilayer configurations, a lossless medium or a lossy medium is considered an upper layer on a metal reflector. The lossless medium has only real RIs (*n*), and the lossy medium has both real and imaginary RI indices (*n*, *k*). In the trilayer configuration, both a lossless medium as an upper layer and a lossy medium as an intermediate layer are located on a metal reflector (Figure 1c). For comparison, three GT resonators with bilayers and a trilayer composed of a lossless medium and/or a lossy medium were designed to have the same resonant wavelength (526 nm).

The bilayered GT resonator with a lossless medium (TiO_2_, 25 nm), despite its rather high n values, by itself cannot provide a strong resonance by multiple interferences with the high reflectivity of a metallic reflector. Therefore, at the target resonant wavelength, this GT resonator rarely exhibits a distinct reflection dip and a group delay peak (Figure 1d). As previously reported [29], the bilayered GT resonator with lossy medium (a-Si, 9 nm) provides a strong resonance by multiple interferences with high complex RIs of the lossy medium on a metallic reflector. So, this GT resonator shows a distinct reflection dip and a narrow GD peak at the target resonant wavelength (Figure 1e). The trilayered GT resonator with lossless medium (SiO_2_, 80 nm) and lossy medium (*P_r_*–Ge, 60 nm) introduced in this work successfully provides multiple interference-induced strong resonance on metallic reflectors even with the low *n* values of the lossless medium and the relatively low complex RIs of the lossy medium. Therefore, the trilayered GT resonator also exhibits a distinct reflection dip and a narrow GD peak at the target resonant wavelength (Figure 1f).

To confirm the advantage of the GT resonator designed with low RIs, we performed optical simulations for each bi-/trilayered structure with several situations of the low RI substance on the top layer. For a thickness variation of a low RI (*n*~1.5) layer, the bilayered GT resonator with a lossless medium is not already under a strong resonance condition, so there is only a change in reflectivity (*R*), and a linear resonant shift is not observed in the bilayered GT resonator (Figure 1g). In the bilayered GT resonator with a lossy medium, the resonance shift was hardly observed with respect to the thickness change of the low RI layer, and the resonance condition deviated above a certain thickness (Figure 1h). Significantly, the trilayered GT resonator showed a linear shift of strong resonance according to the thickness change of the low RI layer (Figure 1i). For the low RI nanoparticles (NPs) locally on the top surface, the bilayered GT resonator with a lossless medium showed no significant differences in the spectrally and spatially electric field distributions at the position of the NPs, with a subtle change in GD (Figure 1j). Even in the bilayered GT resonator with a lossy medium, there was only a slight difference in the position of the NPs in electric field distribution, with a slight shift in the GD peak (Figure 1k). Impressively, the trilayered GT resonator showed a drastic change in the electric field distribution at the location of the NPs and a distinct shift in the GD with high wavelength resolution (Figure 1l).

### 3.2. Design of Trilayered GT Resonators by Impedance Matching Based on the Transmission Line Theory

**Figure 2:**
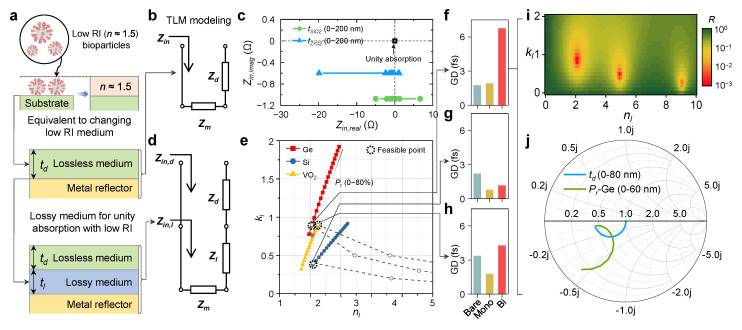
(**a**) Schematic of a design approach for a GT resonator that is sensitive to changes in low RI bioparticles. (**b**) Transmission line models equivalent to bilayer configurations of GT resonators. (**c**) Complex plane plot with a unity absorption point for a bilayer configuration with SiO_2_ (green circle) and ZrO_2_ (blue triangle). (**d**) Transmission line models equivalent to trilayer configurations for designing GT resonators sensitive to changes in low RI bioparticles. (**e**) Solution space for various *t_d_* composed of Ge (red square), Si (navy circle), and VO_2_ (yellow triangle) increases from 0 nm to 100 nm in 5 steps; *P_r_* increases from 0 to 80 in 5% increments. (**f**–**h**) Comparison of GDs of designs with (**f**) Ge, (**g**) VO_2_, and (**h**) Si for bare, mono-, and bilayers. (**i**) Reflectivity contour plot as a function of *n_l_* and *k_l_* at a *t_d_* of 80 nm. (**j**) Impedance trajectory of a realistic design on the Smith chart.

Figure 2a illustrates a GT resonator configuration in a low-index medium as a simple approach to a low-index bioparticle sensor. A situation in which bioparticles are captured on a substrate can be considered equivalent to a change in a low RI medium, such as that of the virus (*n*~1.5) [40]. Therefore, a resonance-based sensing platform for virus detection should be designed to be sensitive to changes in a low RI medium. As a first step toward this approach, Figure 2b shows the transmission line modeling for a bilayered GT resonator with a lossless medium [41], defined by the following equation:

(1) $$Z_{in}{=Z}_{}\frac{Z_{m}{+iZ}_{d}tan\left(\beta_{d}t_{d}\right)}{Z_{d}{+iZ}_{m}tan\left(\beta_{d}t_{d}\right)},$$

$Z_{in}$ is the input impedance of the system, where $\beta_{d}$ is the wavenumber in the low RI medium given by $2{\pi n}_{d}/\lambda$. The low RI medium and metal reflector are converted to a transmission with impedances of $Z_{d}$ (= $Z_{0}{/n}_{d}$) and $Z_{m}$ (= $Z_{0}{/N}_{m}$; $N_{m}{=n}_{m}{+ik}_{m}$), respectively. The *n_m_* and *k_m_* are the RI and extinction coefficient of metal, respectively, and $Z_{0}$ is the impedance of free space. The reflection coefficient r of light incident from the air upon the system is given by

(2) $$r=\frac{Z_{in}{-Z}_{0}}{Z_{in}{+Z}_{0}},$$

and the reflectivity is defined as R= ${\left|r\right|}^{2}$. Consequently, the absorption A in the system is 1-R. Unity absorption occurs when R=0, which in turn leads to the impedance-matching condition, i.e., $Z_{in}{=Z}_{0}$. To fix the values, gold (Au) was selected as the metal reflector, and the low RI medium was set to *n* ~ 1.5, similar to the RI of viruses. Then, the impedance-matching condition was expressed by the following simple equation as a function of the thickness of the dielectric layer ($t_{d}$):

(3) $$tan\left(\frac{{2\pi n}_{d}t_{d}}{\lambda }\right)=i\frac{n_{d}{(N}_{m}+1)}{N_{m}{-n}_{d}^{2}},$$

(4) $$f\left(t_{d}\right)=tan\left(\frac{{2\pi n}_{d}t_{d}}{\lambda }\right)-i\frac{n_{d}{(N}_{m}+1)}{N_{m}{-n}_{d}^{2}},$$

where *n_d_* is the RI of the dielectric layer. As inferred from the simplified equation, the low RI medium has only real values, i.e., $n_{d}$; hence, the unity absorption condition cannot be achieved by adjusting only the real value $t_{d}$ as a variable, under the condition that the imaginary part is not canceled. For practical application, we employed several real materials (SiO_2_ and ZrO_2_) as dielectric layers. Figure 2c shows the complex plane plot for SiO_2_ and ZrO_2_ ($t_{SiO2}$ and $t_{ZrO2}$) at a specific wavelength of 526 nm. As expected, the results show that neither lossless dielectric material approaches close to unity absorption.

**Figure 3:**
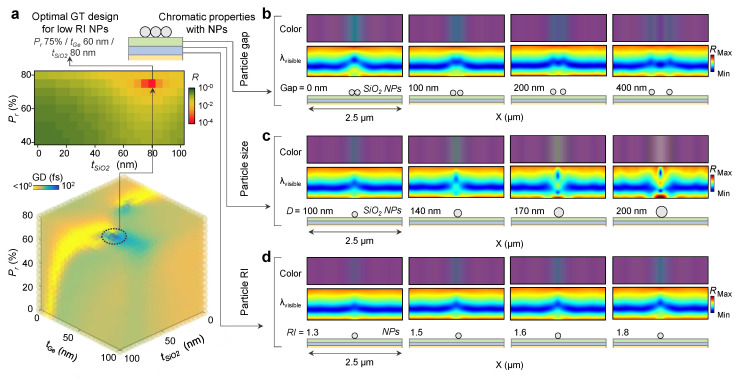
(**a**) GD and reflectivity plots of design parameters for optimal GT design for low RI NPs (**b**–**d**) Chromatic properties with NPs for (**b**) particle gap (0 nm, 100 nm, 200 nm, and 400 nm), (**c**) size (100 nm, 140 nm, 170 nm, and 200 nm), and (**d**) RI (*n* = 1.3, 1.5, 1.6, and 1.8) on the optimal GT design. *λ_visible_* is the visible wavelength range from 400 nm to 800 nm. The green, cyan, and yellow thin films in the schematic view represent SiO_2_, *P_r_*-Ge, and Au, respectively.

As an advanced design approach, a lossy medium capable of canceling the imaginary part under a change of the low RI medium is required to achieve the unity absorption condition. Accordingly, Figure 3d shows a transmission line model equivalent to a trilayer configuration. In the equivalent transmission line model of the trilayer system, outlined in the following equations, $Z_{in,l}$ and $Z_{in,d}$ are the input impedances of the lossy medium and the low RI medium, respectively. The lossy medium is converted to a transmission with impedances of $Z_{l}$ (=$Z_{0}{/N}_{l}$; $N_{l}{=n}_{l}{+ik}_{l}$) [42], where *n_l_* and *k_l_* are the complex RIs of the lossy medium.

(5) $$Z_{in,l}{=Z}_{l}\frac{Z_{m}{+iZ}_{l}tan\left(\beta_{l}t_{l}\right)}{Z_{l}{+iZ}_{m}tan\left(\beta_{l}t_{l}\right)},$$

(6) $$Z_{in,d}{=Z}_{d}\frac{Z_{in,l}{+iZ}_{d}tan\left(\beta_{d}t_{d}\right)}{Z_{d}{+iZ}_{in,l}tan\left(\beta_{d}t_{d}\right)},$$

(7) $$r=\frac{Z_{in,d}{-Z}_{0}}{Z_{in,d}{+Z}_{0}},$$

The impedance-matching condition for unity absorption ($Z_{in,d}{=Z}_{0}$), according to the complex RI, i.e., *n_l_* and *k_l_*, is represented by the solution space with several realistic designs of applicable lossy materials (Ge, Si, and VO_2_) with *P_r_* (Figure 2e and Figure S1, Supplementary Materials for more detailed designs with different RI). Then, these designs were evaluated with the calculated GD values along with the NPs to obtain the optimal design (Figure 2f–h). Among the designs, the optimal structure was determined from the result showing the highest GD value in various situations (Figure 2f). The optimal structure was identified as the solution for the lowest complex RI value in the reflectivity contour plot (Figure 2i). Moreover, the optimal structure was confirmed to achieve impedance matching even in the Smith chart (Figure 2j).

### 3.3. Optimal Modeling Process of the GT Resonator and Colorimetric Visualization

In order to achieve our goal, in this work, we considered low RI materials (i.e., *n*~1.4–1.6), which are observed in the biological materials [40]. According to the calculation result of impedance matching for unity absorption on the low RI sensing layer, it requires a low complex RI of the lossy layer (Figures S2 and S3, Supplementary Materials for more detailed results). Due to the inherent material properties of conventional lossy material (i.e., high complex RI of Ge and Si), the index modulation process is necessary. In this work, we applied *P_r_* on the lossy medium, resulting in a continuously controlled complex RI, and we used SiO_2_ NPs as low RI bioparticles (Figure S4, Supplementary Materials for more detailed results with different RIs). As described in Figure 3a (top), the schematic image and reflectance contour show the structural designing process based on the *P_r_* control of the Ge lossy layer and the thickness of the SiO_2_ dielectric layer. With the parameters (*P_r_* 75% of Ge, *t_Ge_* 60 nm, and *t_SiO2_* 80 nm), the trilayered GT resonator achieves unity absorption. In addition, we confirmed this tendency in the calculation results of GD (Figure 3a, bottom). In that condition, the three-dimensional plot shows the highest GD, which represents an ultrasensitive slow-light condition. Based on the optimal structure, spatial monitoring and colorimetric characterization were performed by finite-difference time-domain (FDTD) calculation. Figure 3b–d show the calculated reflectance contour in visible wavelength (*λ_visible_*) and reflective colors, which have been converted from each reflectance corresponding to the spatial position (Supplementary Note S1, Supplementary Materials for CIE color space and color-matching functions). Figure 3b represents the color distribution and spectrum contour with varying interdistance between the NPs that have fixed diameters (i.e., 100 nm). For the difference gap (0 nm, 100 nm, 200 nm, and 400 nm), in all cases, it shows a color contrast on the location of the NPs; however, for the smaller gap, the resonance wavelength change is stronger (Figures S5 and S6, Supplementary Materials for more results with different RIs). As shown in Figure 3c, the color distribution and spectrum contour were calculated with single NPs that have different diameters (100 nm, 140 nm, 170 nm, and 200 nm). Each case shows different chromatic values and different spectra by growing the diameter. As a final case, Figure 3d shows the calculation results for single NPs that have different RIs (*n* = 1.3, 1.5, 1.6, and 1.8). Due to the strong light–matter interaction with regard to high RI NPs, they show distinct color variation in accordance with the substrate (Figure S7, Supplementary Materials for more results with different RIs). From the process, we find that the chromatic response differs corresponding to the geometrical and physical properties of the target material. Thus, for the robust operation of our trilayered GT resonator, we suggest the representative design for sensing diverse target materials via the numerical calculation process (Table S1, Supplementary Materials for detailed designs).

### 3.4. Visualization Process of NPs and Comparison with Various Substrates

**Figure 4:**
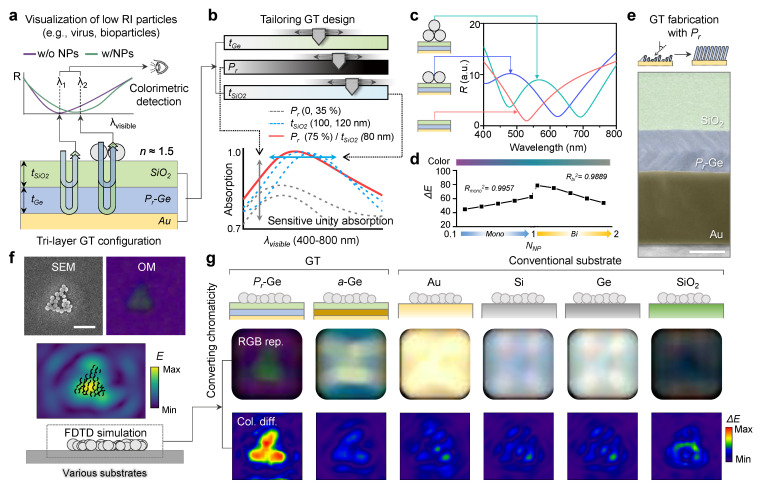
(**a**) Schematic of trilayer configuration of Gires–Torunois (GT) resonator to visualize low refractive index (RI) particles. (**b**) Sensitive unity absorption by adjustment of parameters to tailor GT design. Unity absorption (red line) is achieved only in optimal design (*P_r_* 75% / *t_SiO2_* 80 nm). (**c**) Reflectivity spectra with dip shift according to the morphology of NPs (bilayer, green line; monolayer, blue line; bulk, red line) on GT resonators. (**d**) Strong linear correlations from color difference versus the number of particles in monolayer and bilayer, respectively. *R_mono_^2^* and *R_bi_^2^* are coefficients of determination for linear regression. (**e**) GT resonator fabricated by controlling *P_r_* by glancing angle deposition (GLAD). Scale bar, 50 nm. (**f**) SEM and OM images, calculated E-field distribution of nanoparticle (NP) distribution. Scale bar, 500 nm. (**g**) RGB representation and color difference (*ΔE*) converted from FDTD simulation results for NP distribution on different GT resonators (*P_r_*–Ge and a–Ge) and conventional substrates (Au, Si, Ge, and SiO_2_).

Figure 4a illustrates a trilayered GT resonator for the colorimetric detection and visualization of the viral particles. The schematic reflectance spectrum conceptually shows the resonant wavelength shift by attaching the NPs on the surface. In this section, we considered SiO_2_ NPs as an analyte. Figure 4b represents the tailorable GT design by controlling the thicknesses (*t_Ge_* and *t_SiO2_*) and *P_r_* of the lossy layer (Ge) involved in the complex interference, enabling the optimal design for the strong resonance with unity absorption for a highly sensitive response to the low RI NPs. To maximize the chromatic variation, the resonant wavelength was targeted in the chromatically sensitive range, which has strong spectral sensitivity (Figure S8, Supplementary Materials). Figure 4c shows reflectance spectra for various distributions of NPs. As described in the spectra, the resonance wavelength is slightly shifted upon the geometrical arrangement of NPs (i.e., the gap between NPs and the number of NP layers, see Figure S9, Supplementary Materials). From the calculation result, Figure 4d shows the chromaticity values (i.e., color difference, *ΔE*), corresponding to the particle distribution for mono- and bilayers. Based on this well-classified chromaticity, we modeled a postprocessing algorithm for the visualization of low RI NPs. For experimental confirmation, we applied the *P_r_* on Ge via GLAD [43,44]. Figure 4e shows a cross-sectional transmission electron microscope (TEM) image of the fabricated structure, which represents a porous morphology with tilted nanocolumns (Figure S10, Supplementary Materials for the magnified TEM image). Based on the experimentally realized trilayered GT resonator, we placed SiO_2_ NPs using the immunoassay method (see details in the Materials and Methods Section). In the process, NPs usually formed clusters with mono- or bilayers on the surface, given the Marangoni flow. For NP cluster analysis, we extracted geometrical information of the cluster from scanning electron microscope (SEM) images matched with optical microscopic images. From the extracted information, we made a computational model for numerical calculations; as a result, the strongly confined electric field was observed nearby the NP cluster at the visible range, resulting in a highly sensitive chromaticity change. Finally, we converted the numerical calculation result with chromatic values (color expression and *ΔE*). Figure 4f exhibits the experimental results including the SEM and OM images of the trilayered GT resonator after biosensing, which shows the sharp color contrast with high *ΔE*. For comparison, we performed FDTD simulations with GT structures and conventional substrates by modeling the same NP distribution as the experimental structure. The result of the GT structure with the *P_r_*–Ge layer shows a distinct color in the cluster area similar to the experimental OM image, whereas the cases of an a–Ge intermediate layer and conventional bulk substrate show an unclear color contrast with small *ΔE* (Figure S11, Supplementary Materials for more different results).

## 4. Conclusions

We presented an effective strategy for the colorimetric detection of low RI NPs based on slow-light-induced ultrasensitive chromatic response with a trilayered GT resonator. In this work, we suggested a design flow by modifying the layer configuration and optical constants of a GT resonator with an equivalent transmission line model. The impedance matching was exploited to find design solutions for unity absorption and slow-light conditions. Among the derived designs, the comparatively evaluated optimal design confirmed significant colorimetric sensing performance in various situations of low refractive index substances compared to conventional bilayered GT resonators. In this process, we noted that the trilayered GT resonator sensitive to low RI NPs was successfully configured by modifying the optical constants of the lossy medium by applying *P_r_*. For experimental confirmation, a trilayered GT resonator was fabricated using GLAD, and the low RI bioparticles were locally detected colorimetrically on the surface by the immunoassay process. With the established design flow, we further envision that the trilayer scheme of the GT resonator would be helpful for the multiscale imaging of analytes with various refractive indices and sizes, without special treatments.

## Data Availability

Data presented in this article are available on request from the corresponding author.

## Supplementary Materials

The following supporting information can be downloaded at https://www.mdpi.com/article/10.3390/nano13020319/s1, Figure S1: Reflectance of trilayered GT resonator with SiO_2_ and ZrO_2_, Figure S2: The design rule optimization, Figure S3: Impedance trajectory of a respective realistic design on the Smith chart, Figure S4: Feasible unity absorption point of a GT resonator, Figure S5: Optical characteristics of GT resonator with NPs. Spatial color difference (*ΔE*) of GT resonator with various morphology of NP clusters, Figure S6: The calculated fields and colors with particle gaps, sizes, and refractive indices, Figure S7: Linear correlation of color difference and the number of particles in GT resonator, Figure S8: Chromatically sensitive range in the visible spectrum, Figure S9: Reflectance spectra according to the effective number of NPs, Figure S10: The cross-sectional TEM image of the tri-layered GT resonator composed of SiO2/Pr-Ge/Au (left) and magnified TEM image (right), Figure S11: Calculated colors of the cluster area with different substrates, Table S1: The design specifications of each combination. Reference [45] are cited in the supplementary materials.
