# Supplementary material for: Trilayered Gires–Tournois Resonator with Ultrasensitive Slow-Light Condition for Colorimetric Detection of Bioparticles
Source: Nanomaterials (Basel). 2023 Jan 12;13(2):319. doi: 10.3390/nano13020319 (PMC9865847; doi:10.3390/nano13020319)
Supplement: Supplementary file 1 [file nanomaterials-13-00319-s001.zip › nanomaterials-2148276 supplementary materials.pdf]

nanomaterials

Supplementary Materials

Trilayered Gires-Tournois Resonator with Ultrasensitive Slow-Light Condition for Colorimetric Detection of Bioparticles

Jiwon Kang [1,†], Young Jin Yoo [1,†], Joo Hwan Ko [1], Abdullah Al Mahmud [1], and Young Min Song [1,2,3,*]

[1] School of Electrical Engineering and Computer Science, Gwangju Institute of Science and Technology, 123 Cheomdangwagi-ro, Gwangju 61005, Republic of Korea
[2] Anti-Viral Research Center, Gwangju Institute of Science and Technology, 123 Cheomdangwagi-ro, Gwangju 61005, Republic of Korea
[3] Artificial Intelligence (AI) Graduate School, Gwangju Institute of Science and Technology, 123 Cheomdangwagi-ro, Gwangju 61005, Republic of Korea
* Correspondence: ymsong@gist.ac.kr
† These authors contributed equally to this work.

Supplementary Note S1: CIE color space and color-matching functions

The CIE 1931 color space was adopted to assess the reflectance spectra in the visible wavelength range (400-800 nm) into chromatic values [1]. The reflectance spectra were calculated into chromatic information using the CIE reference primaries *X*, *Y*, and *Z* which represent three primary colors red, green, and blue (*RGB*) as different amounts of the trichromatic additive color model, harnessing the following equations:

$$X=\int M(\lambda)\bar{x}(\lambda)\,d\lambda,\ \ Y=\int M(\lambda)\bar{y}(\lambda)\,d\lambda,\ \ Z=\int M(\lambda)\bar{z}(\lambda)\,d\lambda$$

where, $\bar{x}(\lambda)$, $\bar{y}(\lambda)$, and $\bar{z}(\lambda)$ are color-matching functions, which express numerical descriptions of the chromatic response of the observer. The reflectance spectra in the visible region which were calculated or experimentally measured were multiplied by the spectral power distribution ($M(\lambda)$) over the visible wavelength. In the CIE coordinate, the chromaticity is derived using x and y which are normalized parameters of tristimulus values:

$$x=\frac{X}{X+Y+Z},\ \ y=\frac{Y}{X+Y+Z}$$

The tristimulus values were transformed into the *RGB* values using the following matrix:

$$\begin{bmatrix}R\\G\\B\end{bmatrix}=\begin{bmatrix}0.41847 & -0.15866 & -0.082835\\-0.091169 & 0.25243 & 0.015708\\0.00092090 & -0.0025498 & 0.17860\end{bmatrix}\cdot\begin{bmatrix}X\\Y\\Z\end{bmatrix}$$

The chromatic information of reflectance spectra is predicted or analyzed using these converted *RGB* values.

Also, the color difference values, ΔE and ΔH, derived from the extended color gamut CIE $L^*a^*b^*$, regarding the L^* (lightness from black (0) to white (100)), a^* (chromatic value of green (-) to red (+)), and b^* (chromatic value from blue (-) to yellow (+)), are utilized to quantify and evaluate the colors by the following equations:

$$L^*=116f\left(\frac{Y}{Y_n}\right)\text{-}16,\ a^*=500\left(f\left(\frac{X}{X_n}\right)\text{-}f\left(\frac{Y}{Y_n}\right)\right),\ b^*=200\left(f\left(\frac{Y}{Y_n}\right)\text{-}f\left(\frac{Z}{Z_n}\right)\right)$$

Three parameters X_n, Y_n, and Z_n represent the white point under illuminant D65 conditions with normalization of $Y = 100$ as CIE 1931 XYZ tristimulus values, which is obtained as $X_n = 95.0489$, $Y_n = 100$, and $Z_n = 108.8840$. The function $f(t)$ is depending on the value of t; $f(t)=\sqrt[3]{t}$ for $t > \delta^3$ or $f(t) = \frac{t}{3\delta^2}+\frac{4}{29}$ for other cases, and δ is 6/29. ΔE is derived by the following formula:

$$\Delta E=\sqrt{\left(\Delta L^*\right)^2+\left(\Delta a^*\right)^2+\left(\Delta b^*\right)^2}$$

where,

$$\Delta L^* = L_2^*\text{-}L_1^*,\ \Delta a^* = a_1^*\text{-}a_2^*,\ \Delta b= b_1^*\text{-}b_2^*$$

In addition, another color difference value ΔH was introduced to specify the hue difference for primary colors and greyscales, since the human eye is more sensitive to some areas of color and less sensitive to others. ΔH is derived as:

$$\Delta H=\sqrt{(\Delta E)^2\text{-}\left(\Delta L^*\right)^2\text{-}\left(\Delta C^*\right)^2}$$

where,

$$\Delta C_1^*=\sqrt{a_1^{*2}+b_1^{*2}},\ C_2^*=\sqrt{a_2^{*2}+b_2^{*2}},\ \Delta C^* = C_2^*\text{-}C_1^*$$

We quantified and evaluated the colors and color differences using customized MATLAB (MathWorks, USA) codes that carry out the above equations and relations for both simulated and experimental results.

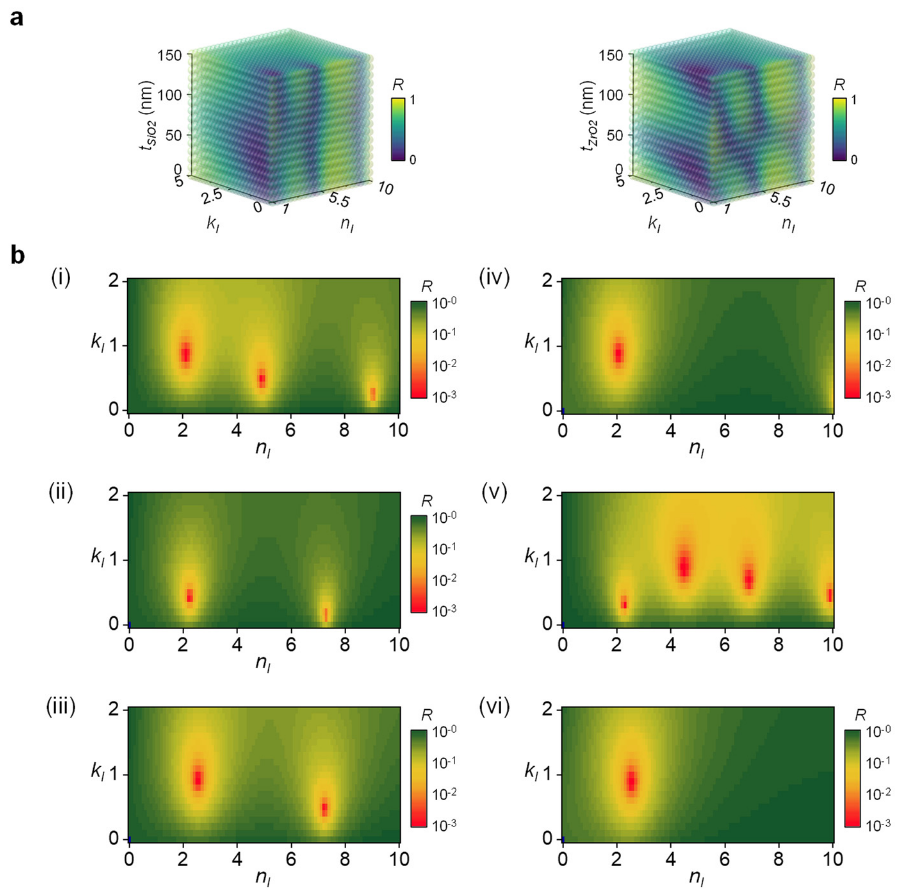

Figure S1: Reflectance of tri-layer GT resonator with SiO_2 and ZrO_2. (a) 3D reflectivity scatters plot of GT resonator with two oxides (SiO_2 and ZrO_2) depending on different refractive indices (n_l), extinction coefficients (k_l), and the thickness of oxide layer (t_{SiO2}). (b) Optimal solution space with local minima of each tri-layer configuration. Reflectivity contour plot with regard to complex refractive index at a specific thickness of oxide layer.

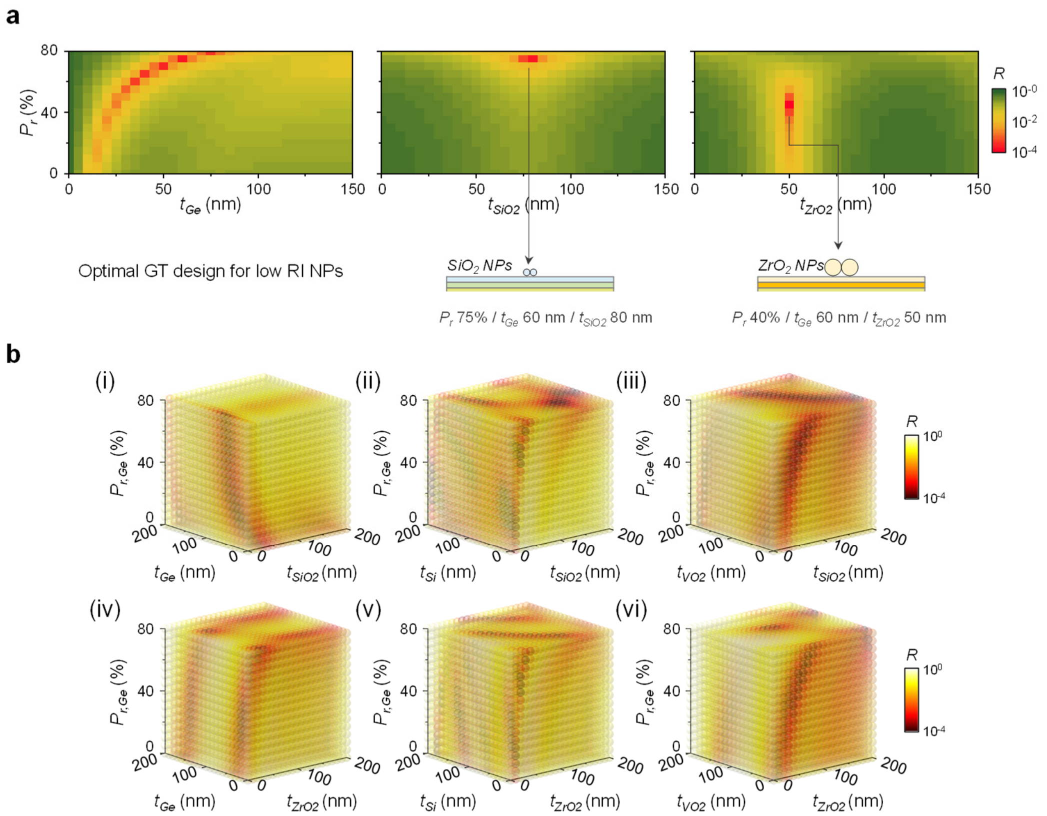

Figure S2: The design rule optimization. (a) Reflectivity contour plot with regard to complex refractive index at a specific thickness of oxide layer. (b) 3D reflectivity scatters plot of GT resonator with six combinations of lossy medium (P_r-Ge, P_r-Si, VO_2) and oxide layers (SiO_2, ZrO_2) depending on different thickness of lossy medium, oxide layer, and porosity(P_r).

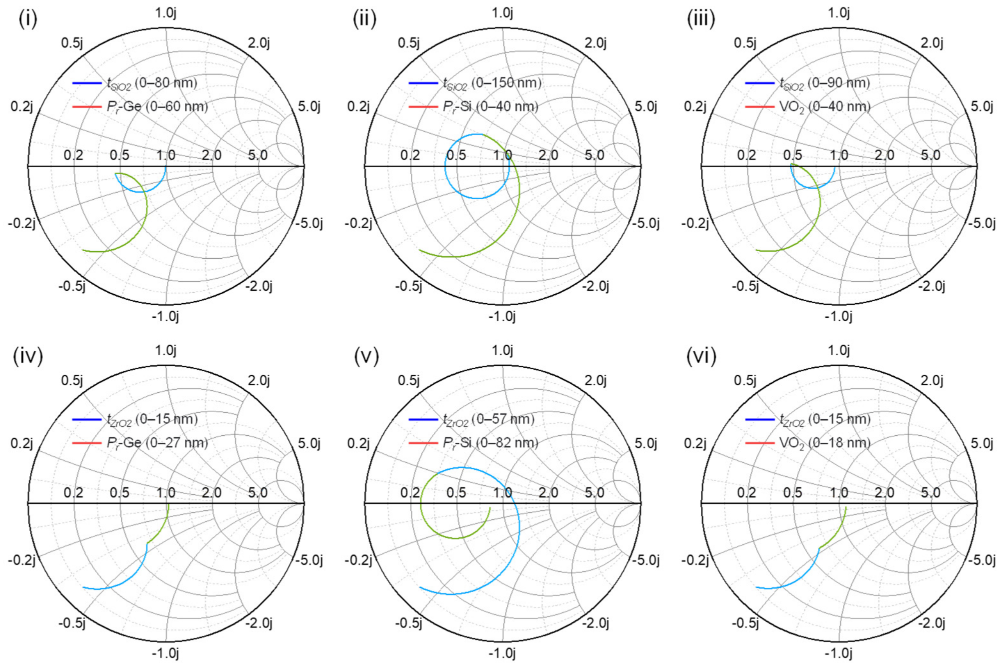

Figure S3: Impedance trajectory of a respective realistic design on the Smith chart.

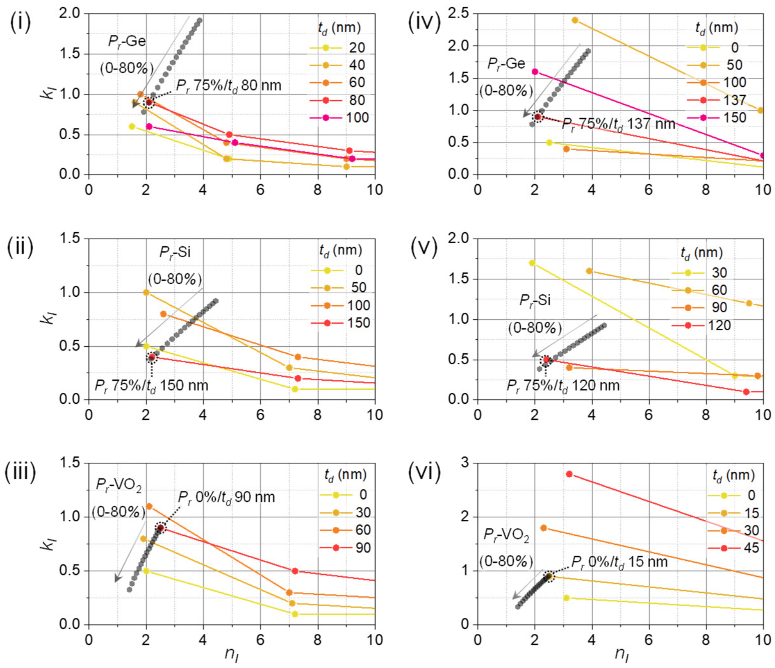

Figure S4: Feasible unity absorption point of a GT resonator. Solution space of local minima with regard to the complex refractive index of GT resonator for various thicknesses of the oxide layer (t_d), and calculated complex refractive index of the respective lossy medium.

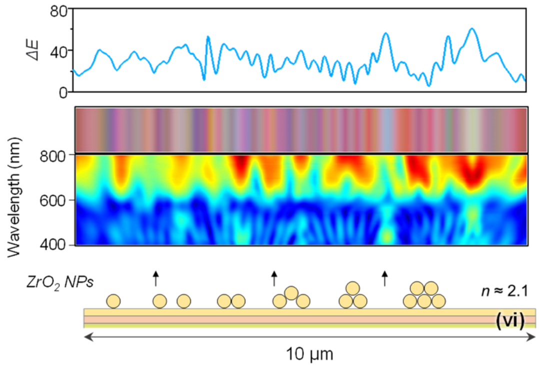

Figure S5: Optical characteristic of GT resonator with NPs. Spatial color difference (*ΔE*) of GT resonator with various morphology of NP clusters. Spatial reflected power spectra (bottom) and color representation (top) are also represented. The illustration shows the morphologies of NPs.

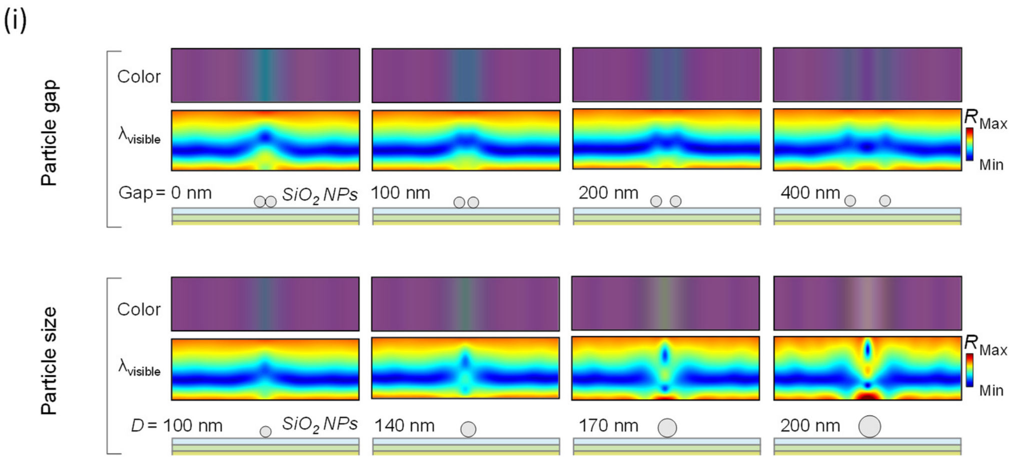

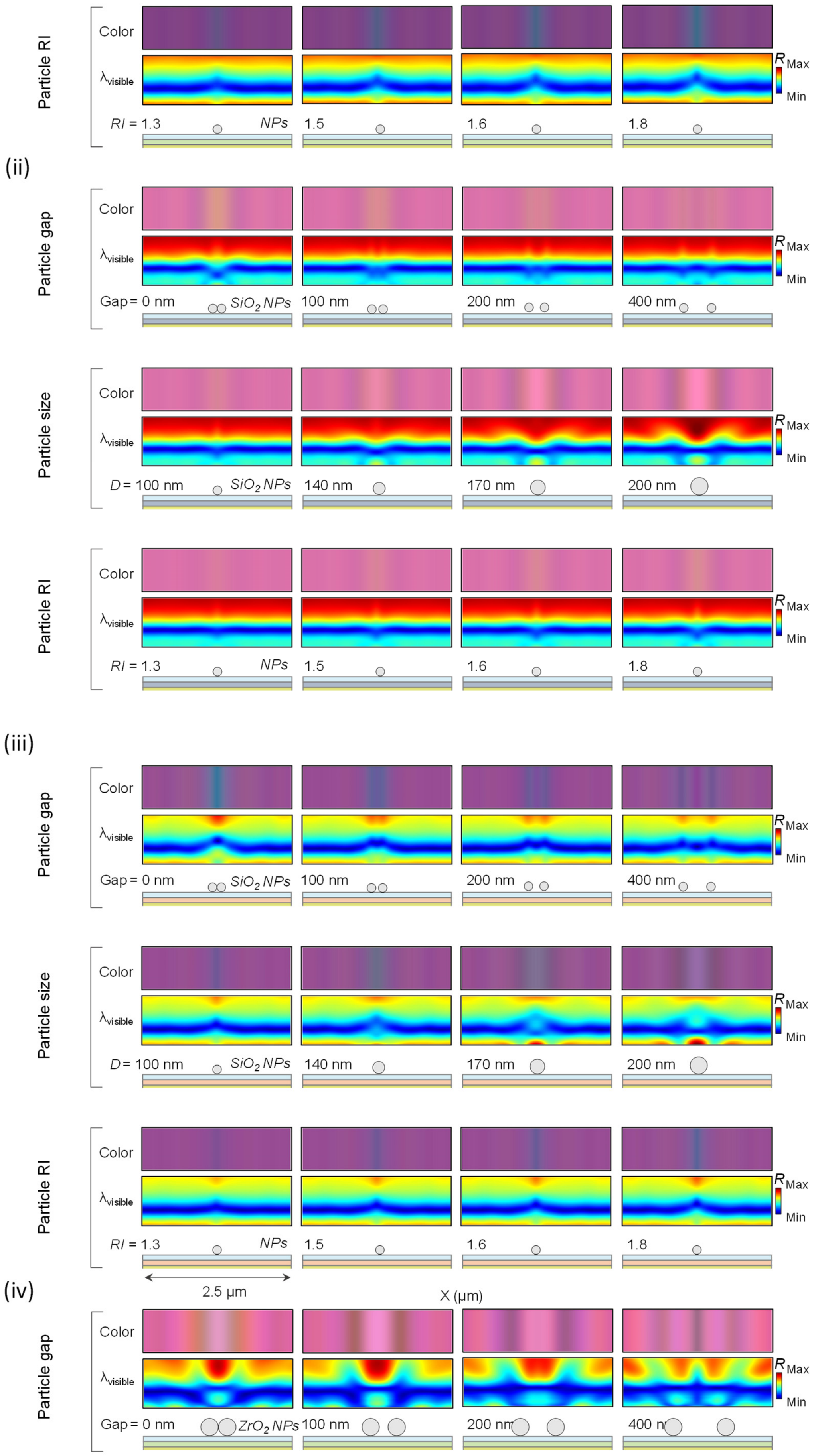
(ii)
Particle RI
Color
λvisible
R Max Min
RI = 1.3 NPs 1.5 1.6 1.8
Particle gap
Gap = 0 nm SiO2 NPs 100 nm 200 nm 400 nm
Particle size
D = 100 nm SiO2 NPs 140 nm 170 nm 200 nm
(iii)
Particle gap
Particle size
Particle RI
2.5 μm
X (μm)
(iv)
Particle gap
Gap = 0 nm ZrO2 NPs 100 nm 200 nm 400 nm

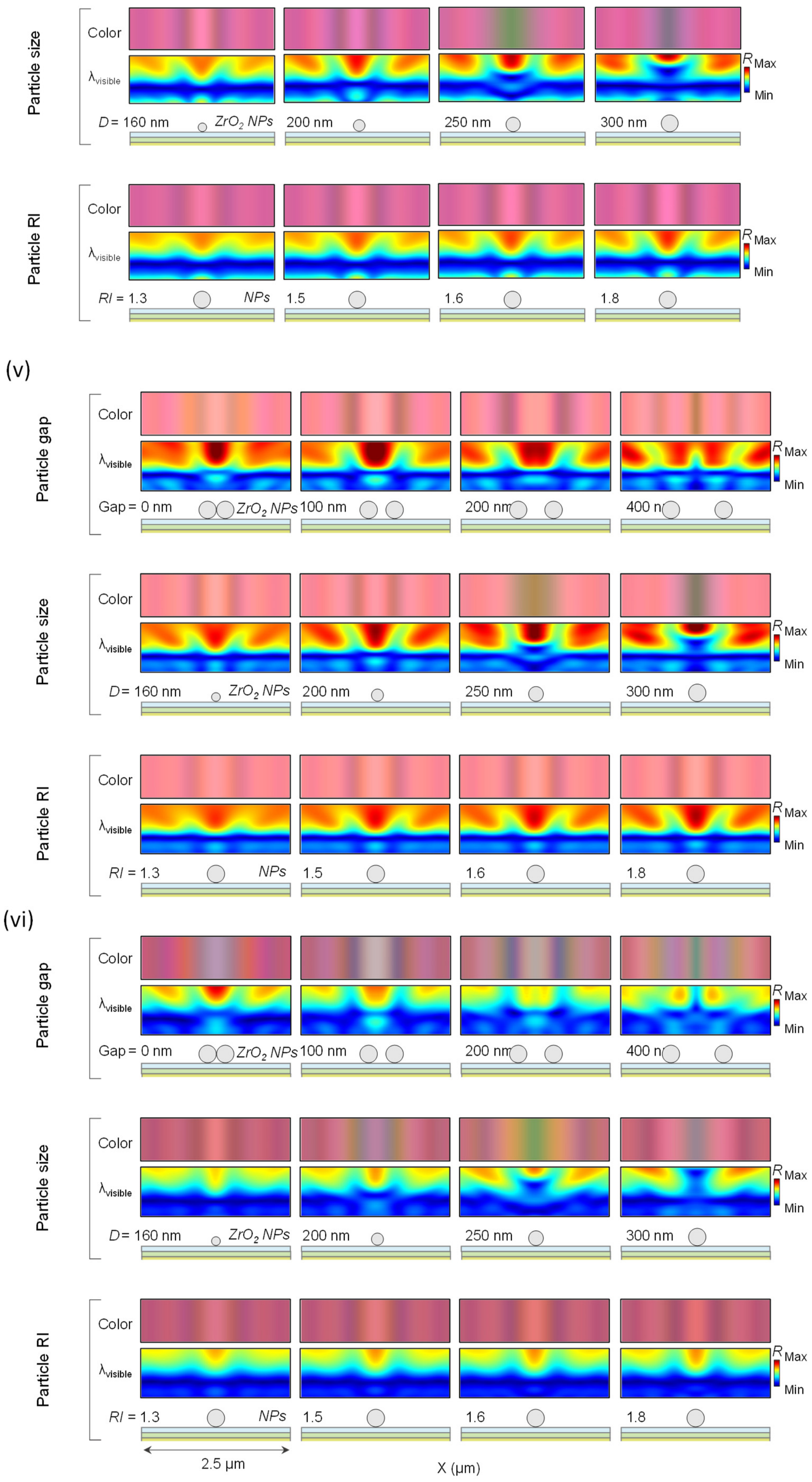

Figure S6: The calculated fields and colors with particle gaps, sizes, and refractive indices.

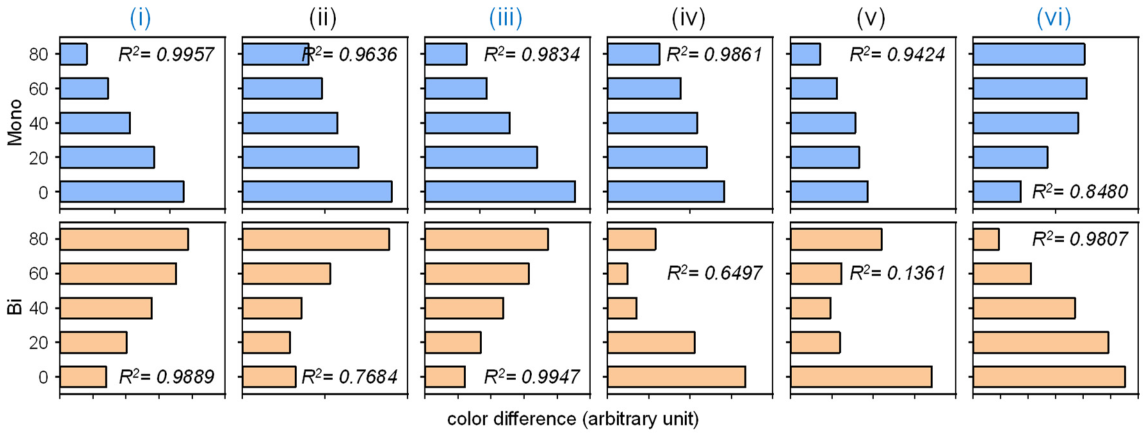

Figure S7: Linear correlation of color difference and the number of particles in GT resonator. Estimated value weighting from the color difference to the number of particles in each design of GT resonator. Linear correlation of mono/bi-layers in (i), (iii), and (vi) is confirmed. R^2 is the coefficient of determination for linear regression.

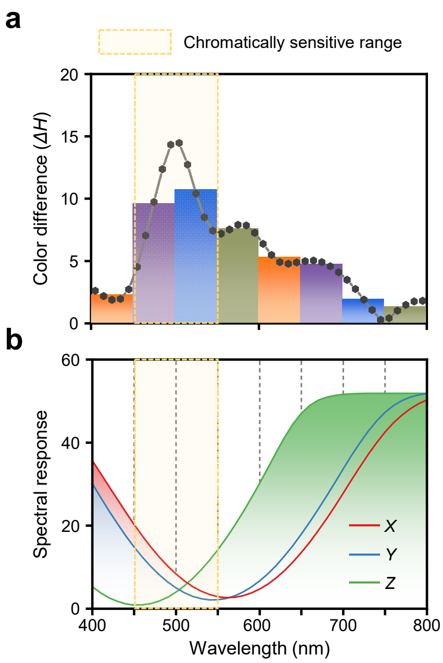

Figure S8: Chromatically sensitive range in the visible spectrum. (a) Calculated color difference to the visible spectrum. (b) Quantified spectral response according to tristimulus function (X, Y, and Z) in human cone cells.

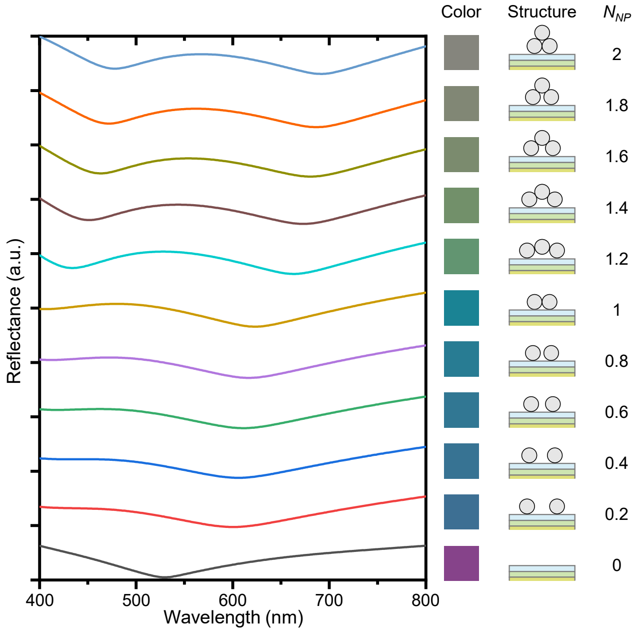

Figure S9: Reflectance spectra according to the effective number of NPs. Calculated reflectance spectra and converted chromaticity of GT resonator according to the physical structure of NPs cluster. N_{NP} is the effective number of NPs.

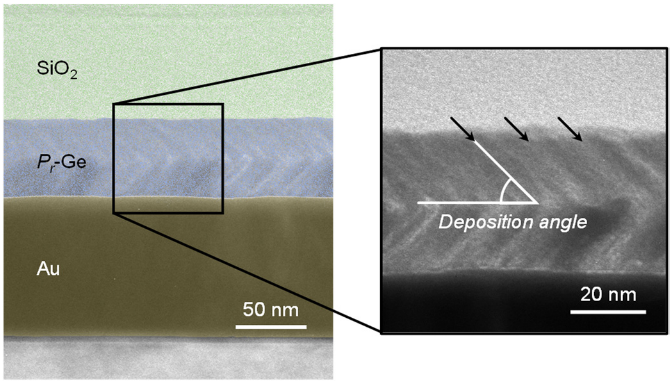

Figure S10: The cross-sectional TEM image of the tri-layered GT resonator composed of SiO_2/P_r-Ge/Au (left) and magnified TEM image (right).

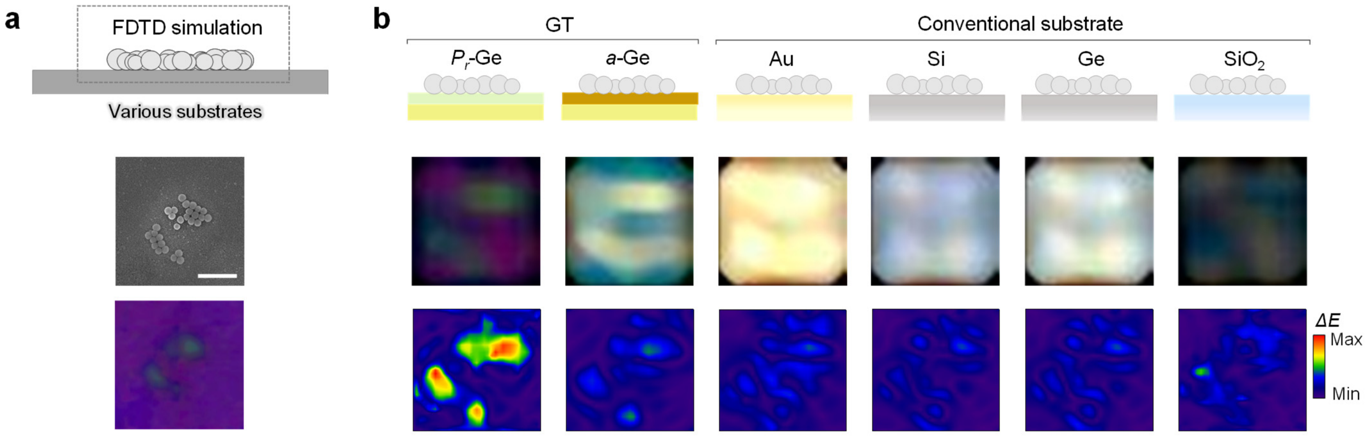

Figure S11: Calculated colors of the cluster area with different substrates. (a) Two NPs cluster formation of ground truth SEM and OM images. They modeled in CAD and calculated mounting on

various substrates via the FDTD method. (b) Simulated colors and color differences of diverse substrates were obtained using the FDTD. The color differences were calculated from the substrate color. Scale bar, 500 nm.

Table S1: The design specifications of each combination.

Design	Lossy medium	Low RI medium
(i)	Ge, P_r 75% / 60 nm	SiO_2 / 80 nm
(ii)	Si, P_r 75% / 40 nm	SiO_2 / 150 nm
(iii)	VO_2, P_r 0% / 40 nm	SiO_2 / 90 nm
(iv)	Ge, P_r 75% / 27 nm	ZrO_2 / 15 nm
(v)	Si, P_r 75% / 82 nm	ZrO_2 / 57 nm
(vi)	VO_2, P_r 0% / 18 nm	ZrO_2 / 15 nm

References

1. Fairman, H.S.; Brill, M.H.; Hemmendinger, H. How the CIE 1931 color-matching functions were derived from Wr ight-Guild data. *Color Res. Appl.* **1998**, *22*, 11–23.
